# Cromolyn prevents cerebral vasospasm and dementia by targeting WDR43

**DOI:** 10.3389/fnagi.2023.1132733

**Published:** 2023-04-13

**Authors:** Xingqiao Wang, Fanqiang Kong, Zengbin Lin

**Affiliations:** Emergency Department, The Affiliated Yantai Yuhuangding Hospital of Qingdao University, Yantai, China

**Keywords:** aortic aneurysm (AA), cerebral vasospasm (CV), cromolyn, cognitive impairment, dementia, aging, neurovascular markers, subarachnoid hemorrhage (SAH)

## Abstract

**Background:**

Cerebral vasospasm (CV) can cause inflammation and damage to neuronal cells in the elderly, leading to dementia.

**Purpose:**

This study aimed to investigate the genetic mechanisms underlying dementia caused by CV in the elderly, identify preventive and therapeutic drugs, and evaluate their efficacy in treating neurodegenerative diseases.

**Methods:**

Genes associated with subarachnoid hemorrhage and CV were acquired and screened for differentially expressed miRNAs (DEmiRNAs) associated with aneurysm rupture. A regulatory network of DEmiRNAs and mRNAs was constructed, and virtual screening was performed to evaluate possible binding patterns between Food and Drug Administration (FDA)-approved drugs and core proteins. Molecular dynamics simulations were performed on the optimal docked complexes. Optimally docked drugs were evaluated for efficacy in the treatment of neurodegenerative diseases through cellular experiments.

**Results:**

The study found upregulated genes (including WDR43 and THBS1) and one downregulated gene associated with aneurysm rupture. Differences in the expression of these genes indicate greater disease risk. DEmiRNAs associated with ruptured aortic aneurysm were identified, of which two could bind to THBS1 and WDR43. Cromolyn and lanoxin formed the best docking complexes with WDR43 and THBS1, respectively. Cellular experiments showed that cromolyn improved BV2 cell viability and enhanced Aβ42 uptake, suggesting its potential as a therapeutic agent for inflammation-related disorders.

**Conclusion:**

The findings suggest that WDR43 and THBS1 are potential targets for preventing and treating CV-induced dementia in the elderly. Cromolyn may have therapeutic value in the treatment of Alzheimer’s disease and dementia.

## 1. Background

Recent studies have primarily focused on investigating the cognitive decline that occurs following stroke, with particular emphasis on hemorrhagic cerebrovascular disease, which has received comparatively less attention than its ischemic counterpart (Petridis et al., 2017; Sharma, 2020). Aneurysmal subarachnoid hemorrhage (aSAH) is associated with a high mortality and disability rate, and survivors often experience cognitive deficits and diminished functioning, significantly impacting their long-term quality of life and productivity (Etminan and Macdonald, 2017). Despite some studies identifying genetic determinants of cerebral vasospasm (CV), the genetic mechanisms underlying CV following aSAH and its potential therapeutic targets have yet to be fully elucidated (Ducruet et al., 2010). To develop preventive and therapeutic strategies for dementia resulting from CV, it is necessary to examine the genetic basis for dementia caused by CV following aSAH.

Individuals who survive aSAH have an elevated risk of developing dementia, and early targeted treatment can significantly improve prognosis (Hasan et al., 2020). Delayed cerebral ischemia (DCI) resulting from CV is a significant predictor of dementia following aSAH (Geraghty and Testai, 2017; Eagles et al., 2019). The underlying cause of CV after aSAH and effective therapeutic targets for CV remain uncertain, emphasizing the need to explore the mechanisms driving its occurrence for improved patient prognosis.

Cerebral vasospasm is a major cause of death and neurodegenerative disease in aSAH patients, and several pharmacological approaches may be effective in preventing and treating CV. However, further investigation is necessary to assess the efficacy of drugs in treating CV and neurodegeneration after aSAH (Akkaya et al., 2020). Developing drugs for neurodegeneration requires identifying the correct target genes and biomarkers (Walker et al., 2018; Athar et al., 2021). Multi-omic bioinformatic tools have emerged as effective methods for identifying biomarkers (Lin et al., 2017; Badhwar et al., 2020). Transcriptome-wide analyses offer a global overview of the cell state and how it changes after disease or treatment (Paananen and Fortino, 2020), while single-cell RNA sequencing (scRNA-seq) enables the analysis of transcriptomes at the single-cell level, circumventing intercellular randomness (Hu et al., 2020; Lam et al., 2020; Wu et al., 2021; Chen et al., 2022a,b; Luo et al., 2022). Single-cell sequencing can be used to understand the development of neurodegenerative diseases at the single-cell level (Wang et al., 2022). Thus, both transcriptomic analysis and scRNA-seq can elucidate the functions of cells during disease development, the molecular mechanisms underlying disease development, and biomarkers associated with disease development, thereby enabling more precise therapeutic development (Shalek and Benson, 2017; Yuan et al., 2017; Sawada et al., 2018). These bioinformatic tools can facilitate the development of new drugs and identification of new therapeutic targets (Matthews et al., 2016; Koscielny et al., 2017).

Understanding the pathogenesis of aortic aneurysm (AA) is essential for preventing and treating subarachnoid hemorrhage (SAH) caused by aneurysms of the brain aorta (Tawk et al., 2021). AA is characterized by permanent dilation of the arteries to 1.5 times their normal size, making them prone to rupture or dissection (Malecki et al., 2020). AA is a chronic degenerative disease involving the breakdown and loss of the structural extracellular matrix (ECM) of the aortic wall, leading to progressive thinning, weakness and eventual rupture of the vessel wall (Wang and Jia, 2020; Dahal et al., 2022). Chronic inflammatory infiltration, degradation of collagen and elastin, and other pathological changes are also involved (Wang and Jia, 2020; Dahal et al., 2022). AA is a life-threatening condition, with approximately 150,000–200,000 deaths reported worldwide annually, leading to a significant socioeconomic burden and causing great suffering to the families of patients (Golledge, 2019; Wen et al., 2020; Ibrahim et al., 2021). While surgical repair is the primary treatment for preventing AA rupture, it has limited indications and does not significantly reduce mortality (Sweeting et al., 2017; Wanhainen et al., 2019; Ibrahim et al., 2021). Therefore, identifying key biomarkers associated with AA and understanding the role of peripheral blood erythrocytes in its development is important.

Previous studies have shown that AA pathogenesis involves the contribution of peripheral red blood cells (RBCs), resulting in endothelial cell damage and the adhesion of RBCs, white blood cells, and platelets to the AA wall (Zhou et al., 2018; Sun et al., 2019). Furthermore, RBC breakdown products have been linked to CV in patients with SAH, leading to neurodegenerative diseases (Bi et al., 2019; Wang et al., 2021). Although inflammatory infiltration, matrix homeostasis, and tissue remodeling in the arterial wall have been investigated previously, there is a lack of intensive research in this area (Zhou et al., 2018; Sun et al., 2019). We hypothesize that further research on AA may reveal potential causes of post-SAH CV.

The goal of this study was to identify potential therapeutic targets for CV that occurs after SAH. To achieve this, we conducted single-cell sequencing and whole-transcriptome analysis, and used molecular docking to identify Food and Drug Administration (FDA)-approved drugs that could be used to treat post-SAH CV and prevent secondary dementia. We then performed *in vitro* assays to verify the efficacy of the identified drugs.

This study aims to contribute to the current understanding of the pathogenesis of post-SAH CV and identify potential therapeutic targets for the treatment and prevention of secondary dementia.

## 2. Materials and methods

### 2.1. Transcriptomic data extraction from patients with cerebral vasospasm (CV) following rupture of aortic aneurysm

The transcriptomic data of patients with CV and AA were extracted from two datasets in the Gene Expression Omnibus (GEO) database (GSE37924 and GSE13353, respectively) available in the NCBI PubMed database. These datasets were selected based on their relevance to the study of dementia and their availability in the NCBI PubMed database. The CV dataset GSE37924 included 34 samples from patients with cerebral artery spasm after subarachnoid hemorrhage and 30 peripheral blood erythrocyte samples, while the GSE13353 dataset included 11 aortic wall tissue samples from patients with ruptured AA and eight samples from patients with unruptured AA. Differentially expressed genes (DEGs) were identified with a corrected *P*-value of 0.05 and a |log_2_FC (fold change)| value greater than 0.58. Volcano plots were used to visualize DEGs, and Venn diagrams were used to determine upregulated and downregulated genes in both datasets. The Gene Set Enrichment Analysis (GSEA) was performed using the “(human) hallmark gene sets” module.

### 2.2. Transcriptomic data extraction of patients with ruptured aortic aneurysms

The GEO database was searched for transcriptomic data related to ruptured AA in Homo sapiens using the keywords “aortic aneurysm rupture” and “miRNA.” The resulting dataset GSE161870 included two samples from patients with ruptured AA and two samples from patients with unruptured AA. The differentially expressed miRNAs (DEmiRNAs) between the ruptured AA and control groups were identified using R package with screening criteria of *P*-value less than 0.05 and a log_2_FC greater than 0.58 or less than −0.58. A volcano map and heat map were constructed to visualize the DEmiRNAs. The predicted target genes of DEmiRNAs were obtained by mapping them to DEGs from microarray analysis based on the negative correlation between miRNAs and mRNAs. Cytoscape (version 3.8.0) was used to construct a model of the miRNA–mRNA interaction network.

### 2.3. Pre-processing and analysis of variance of single-cell data of patients with AA rupture

The single-cell sequencing data of patients with arterial lesions were extracted from the GSE166676 dataset in the GEO database. Four tissue samples with AA lesions and two normal arterial wall tissues were included. Quality control analysis was performed using Limma, Seurat, Dplyr, and Magrittr packages in R to filter and assess cells based on the following conditions: cells with expression of more than 200 genes, those with less than 10,000 genes, and those with less than 20% of mitochondrial genes were retained. Principal component analysis (PCA) was used to identify the component with the highest variance, and the top 20 principal components were selected for t-distributed stochastic neighbor embedding (tSNE) and uniform manifold approximation and projection (UMAP) clustering analysis with a resolution of the clustering parameter set to 2.0. Cell clusters were annotated based on gene expression and the average expression of the marker genes. DEGs between the AA and control groups were identified using DESeq2 and Wilcoxon test, and the heatmap function was used to visualize these DEGs.

### 2.4. Identification of differentially expressed miRNAs, genes, and protein-protein interaction (PPI) network

To identify the differentially expressed miRNAs and genes associated with AA rupture occurring after CV events, we used the Venn R package to perform intersection analysis and generate co-expression Venn diagrams. The STRING database^1^ was used to map and analyze the interaction networks of intersecting proteins. The constructed networks were imported into Cytoscape to construct and visualize a protein–protein interaction (PPI) network of DEmiRNAs and DEGs associated with AA rupture occurring after CV.

### 2.5. Identification of hub genes and construction of diagnostic models

We used the interactivenn tool^2^ to plot a Venn diagram for demonstrating target genes associated with AA rupture occurring after CV. The intersections in the results were considered potential core target genes associated with AA rupture. Receiver operating characteristic (ROC) curves and risk factor curves were plotted to evaluate the area under the curve (AUC) values, sensitivity and specificity of the core genes.

### 2.6. Functional and pathway enrichment analyses

We used the scatterplot3d and clusterProfilerGO.R packages along with Perl language to perform Gene Ontology (GO) functional annotation and Kyoto Encyclopedia of Genes and Genomes (KEGG) pathway enrichment analyses of the core genes. A *P*-value of less than 0.05 was considered significant.

### 2.7. Analysis of hub protein sensitivity

Virtual molecular docking is an effective approach for identifying potential drug candidates. To evaluate the possible biological activity of a compound, we docked FDA-approved drugs from the ZINC database with the core proteins, whose PDB files were downloaded from the PDB database.^3^ We removed water molecules from receptor protein-ligand complexes and used the Gasteiger-Marsili method to assign atomic charges to amino acid residues. The LibDock module of Discovery Studio 2019 was used for docking, and the active pockets, we used the AutoDockTools (version 1.5.6) software. The docking results were visualized in 3D and 2D to evaluate the reliability of the ligand-receptor complexes.

### 2.8. Molecular dynamics simulation

Molecular dynamics (MD) simulations were performed to investigate the stability, kinetic properties, and strength of protein-drug binding. The two best docked complexes from pre-model screening were selected for MD analysis. MD simulations were conducted using the DS2019 simulation module with the Charmn36 force field on FDA-approved drugs. The structural behavior of atoms and molecules was analyzed as time-based functions. Root mean square deviation (RMSD) values and the number of intermolecular hydrogen bonds were used to determine the stability and strength of protein-drug binding.

The docked complexes were solvated in a truncated octahedral periodic box with an eight-molecule water buffer around the core protein water molecules. The Simulation tool was used to neutralize the system charge by adding counteracting ions, including sodium and chloride ions to neutralize the surface charge of the structure. The steepest descent method was used to eliminate van der Waals interactions and hydrogen bonds between water molecules and the docked complex in 50,000 steps. The temperature of the system was gradually increased to 310 K while maintaining a constant volume of 500 ps and establishing equilibrium at a constant pressure of 500 ps. Simulations were performed for 100 ns at 310 K. The Analyze Trajectory module was used to evaluate the interactions between non-bonded molecules. Results were saved at 1 ns intervals for analysis.

### 2.9. Cell culture and RNA interference

The microglia cell line HMC3 (CRL-3304) was obtained from American Type Culture Collection (ATCC). The cells were cultured in DMEM supplemented with 10% fetal bovine serum, 1% L-glutamine, and 1% penicillin/streptomycin in a 5% CO_2_ incubator at 37^°^C. The final concentrations of cromolyn (AZTerapies, MA, USA) were prepared by diluting it with DMEM as described previously (Wang et al., 2021). Cells were activated by treating them with 0.3 g/mL of TNF daily for 24 h (Wang et al., 2021).

shCtl and shWDR43 lentiviruses were transfected into 293T cells (Bi et al., 2019; Sun et al., 2022; Di et al., 2023). The cells were transfected thrice to achieve efficient knockdown. Infected cells were selected with puromycin for 48 h starting from 24 h post-infection. The cells were harvested using TRIzol or sodium dodecyl sulfate (SDS) loading buffer for RNA or protein extraction, respectively.

### 2.10. Western blotting

Protein concentrations from RIPA-lysed cells were determined using the BCA protein assay kit (KeyGEN BioTECH, Jiangsu, China). Extracted proteins were separated by SDS-PAGE and transferred onto PVDF membranes (Millipore, Billerica, MA, USA). Membranes were blocked using 5% skim milk powder at room temperature for 1 h and incubated overnight at 4^°^C, with primary antibodies. The following day, membranes were washed thrice with TBST and incubated with secondary antibodies for 1 h at room temperature. Protein bands were detected using an ECL system (Biotool, Switzerland), and images were semiquantitatively analyzed using ImageJ software.

### 2.11. Quantitative reverse transcription polymerase chain reaction (qRT-pCR)

Total RNA was extracted using the Quick-RNA MiniPrep Kit (Zymo Research, CA, USA) and reverse transcribed using the High-Capacity cDNA Reverse Transcription Kit (Thermo Fisher Scientific, MA, USA). Quantitative PCR was performed with the following conditions: 2 min at 95^°^C (Phase 1), 40 cycles alternating between 95^°^C for 20 s, 60^°^C for 30 s and 70^°^C for 30 s (Phase 2), and eventually 65^°^C for 5 s. The Cq value of GAPDH was subtracted from the Cq value of target genes to calculate fold change. Normalized gene expression values were compared with those of untreated control samples.

### 2.11. Cell viability assay

Cell viability was assessed using the CCK-8 assay (Sigma, Shanghai, China) according to the manufacturer’s instructions. BV2 cells (5,000 cells/well) were seeded in 96-well plates and cultured overnight at 37^°^C. The cells were then treated with cromolyn for 48 h at 37^°^C. After drug treatment, the cells were incubated with CCK-8 reagent (10 mL) for 2 h, and absorbance was measured at 450 nm using a microplate reader (Bio-Rad Laboratories, Hercules, CA, USA).

### 2.12. Amyloid-beta uptake assay

BV2 cells were cultured in DMEM supplemented with 10% fetal bovine serum, 2 mM L-glutamine and 1% penicillin (Life Technologies, CA, USA) and treated with cromolyn (10, 100, and 1 mM) for 16 h. Subsequently, the cells were incubated with 2 μg/ml Aβ42 (AnaSpec) in serum-free DMEM medium for 3 h. The cells were washed with PBS and lysed with RIPA lysis buffer (EMD Millipore, Darmstadt, Germany); Halt Phosphatase Inhibitor Cocktail (Thermo Fisher Scientific, MA, USA) and 2 mM 1,10-phenanthroline (Sigma, Shanghai, China). The cells were centrifuged at 12,000 g and 4^°^C for 15 min, and the supernatants were collected. Aβ42 levels in the supernatant were assessed using a BCA assay kit (Pierce) and normalized to the total protein concentration.

### 2.13. Statistical analysis

Data from at least three independent experiments were expressed as the mean and standard deviation. GraphPad Prism 6.0 was used for data analysis. Differences between groups were estimated using one-way ANOVA and *t*-tests, with a *P*-value of <0.05 considered significant.

## 3. Results

### 3.1. Comprehensive analysis of differentially expressed genes in CV caused by ruptured aortic aneurysms

**FIGURE 1:**
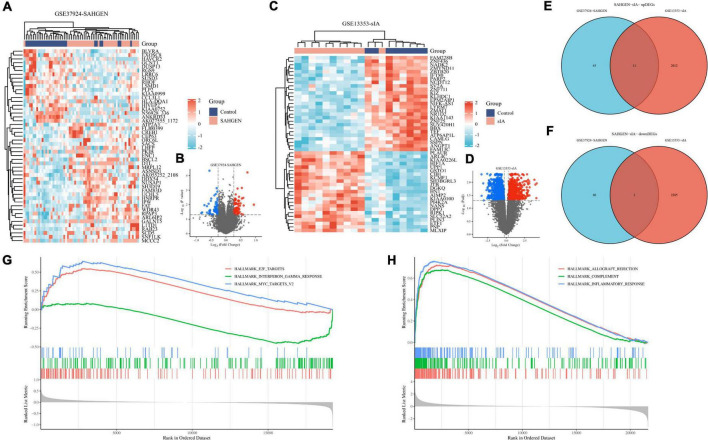
Transcriptomic analysis of patients with ruptured abdominal aortic aneurysms (AAs) and cerebral vasospasm (CV). **(A)** Differentially expressed genes associated with AA rupture identified from erythrocyte samples in the GSE37924 dataset. **(B)** Differentially expressed genes associated with AA rupture identified from erythrocyte samples in the GSE13353 dataset. **(C)** Differentially expressed genes associated with AA rupture identified from erythrocyte samples in the GSE37824 dataset. **(D)** Volcano plot of differentially expressed genes in the GSE13353 dataset (red represents upregulated gene expression, while blue represents downregulated gene expression). **(E)** Venn diagram demonstrating the common upregulated genes in both sample types. **(F)** Venn diagram demonstrating the common downregulated genes in both sample types. **(G)** Graph demonstrating the results of gene set enrichment analysis (GSEA) for all samples. **(H)** Enriched GSEA pathways in all samples.

We performed a comprehensive analysis of gene expression in peripheral blood erythrocytes and aortic wall samples from patients with cardiovascular (CV) pathology caused by ruptured aortic aneurysms. We identified 76 and 61 differentially expressed genes (DEGs) in the GSE37924 dataset and 2286 downregulated and 2023 upregulated genes in the GSE13353 dataset, respectively (Figures 1A–D). After intersecting the two sets of DEGs, we identified 11 upregulated genes (*WDR43, BZW2, SPP1, PHC2, ZNF746, NUSAP1, PLTP, SDCCAG3, TLR5, RFC5, and THBS1*) and 1 downregulated gene (*RGS9*) (Figures 1E, F). gene set enrichment analysis (GSEA) showed that compared to genes associated with unruptured aortic aneurysms, those associated with ruptured aortic aneurysms were significantly enriched in pathways related to E2F targets, interferon-gamma response, MYC target V2, mTORC1 signaling, glycolysis, and epithelial mesenchymal transition in peripheral blood erythrocytes (|NES| ≥ 1.75, *P* < 0.05, Figure 1G). Additionally, genes associated with ruptured aortic aneurysms in aortic wall tissue were significantly enriched in pathways related to allograft rejection, complement cascade, inflammatory response, interferon-gamma response, tumor necrosis factor alpha (TNF-α) signaling *via* NF-κB, and IL6-JAK-STAT3 signaling (|NES| ≥ 2.00, *P* < 0.05, Figure 1H). This study identified differentially expressed genes in patients with cardiovascular pathology caused by ruptured aortic aneurysms and found enrichment in pathways related to immune responses, signaling pathways, and metabolic processes.

### 3.2. Analysis of miRNA data

We also performed an analysis of differentially expressed miRNAs (DEmiRNAs) using the GSE161870 dataset, which contained 406 DEmiRNAs. Of these DEmiRNAs, 213 were upregulated and 193 were downregulated, as determined by heat maps and volcano plots (Supplementary Figures 1A, B), with red indicating upregulation and blue indicating downregulation. To identify potential target mRNAs for these DEmiRNAs, we utilized miRDB, miRTarBase, and TargetScan prediction tools, identifying 9332 mRNAs for upregulated miRNAs and 7517 mRNAs for downregulated miRNAs (Supplementary Figures 1C, D). Specifically, our analysis of the GSE161870 dataset allowed for the identification of a comprehensive list of potential target mRNAs associated with differentially expressed miRNAs. Our findings provide insight into potential molecular mechanisms underlying the observed changes in miRNA expression and may have implications for further research into the diagnosis and treatment of related diseases.

### 3.3. Transcriptomic heterogeneity analysis of single cells in AA samples

**FIGURE 2:**
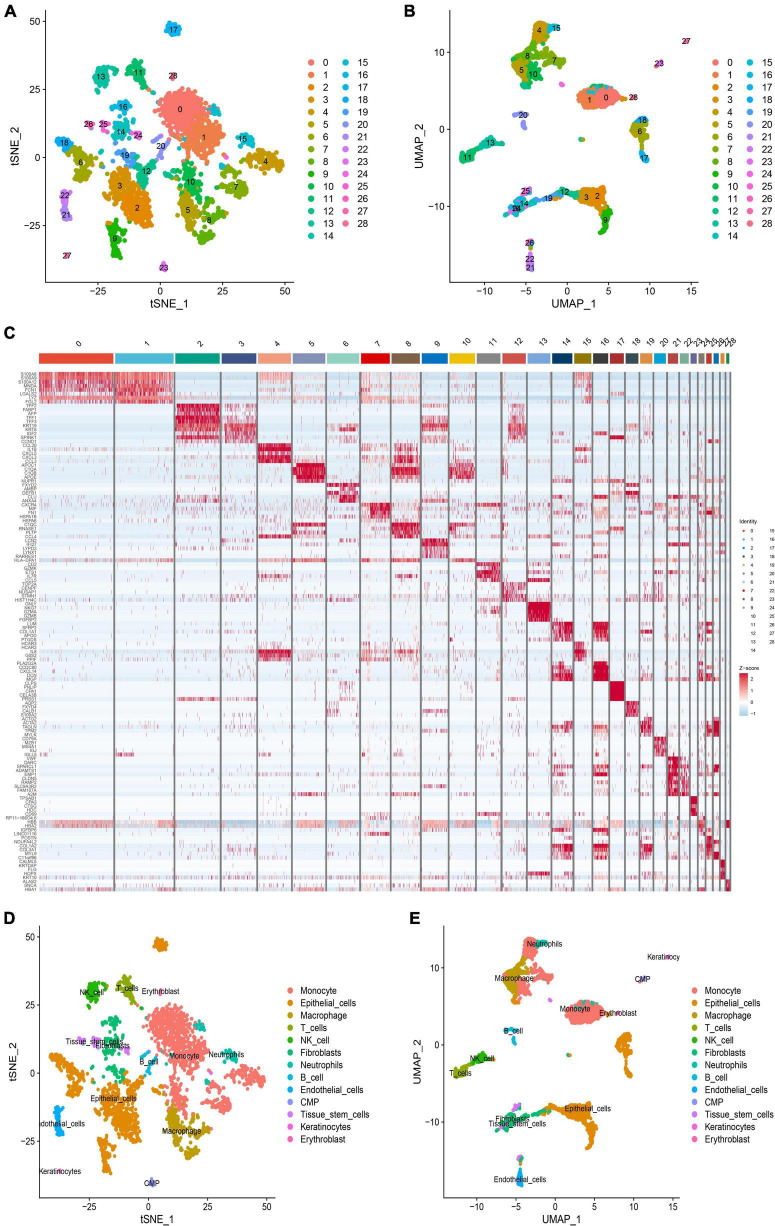
Heterogeneity analysis of ruptured aortic aneurysms. **(A)** Distribution map of t-distributed stochastic neighbor embedding (tSNE) clusters. **(B)** Distribution map of t-distributed stochastic neighbor embedding (tSNE) clusters. **(C)** Heat map showing the expression levels of 28 subgroup markers. **(D)** Cell annotation on the tSNE plot. **(E)** Uniform manifold approximation and projection (UMAP) plots of cell annotation.

The scRNA-seq dataset (GSE166676) comprised 3,100, 2,332, 2,497, 758, 2,209, and 3,196 cells in the AA1, AA2, AA3, AA4, Con1, and Con2 groups, respectively. These cells were integrated and clustered into 28 distinct cell clusters using tSNE and UMAP descending clustering (PC = 20; resolution = 2.0) (Figures 2A, B). To determine cellular identities within each cluster, we estimated the average expression of known marker genes (Figure 2C) and annotated each cluster accordingly. Our analysis revealed the presence of various cell types, including monocytes (Clusters 0, 1, 4, 7, and 10), epithelial cells (Clusters 2, 3, 6, 9, 12, 17, and 18), macrophages (Clusters 5 and 8), T cells (Cluster 11), NK cells (Cluster 13), fibroblasts (Clusters 14, 16, and 19), neutrophils (Cluster 15), B cells (Cluster 20), endothelial cells (Clusters 21 and 22), CMP cells (Cluster 23), tissue stem cells (Clusters 24, 25, and 26), keratinocytes (Cluster 27), and erythroblasts (Cluster 28) (Figures 2D, E).

Furthermore, we utilized the Wilcoxon test and DESeq2 to identify differentially expressed genes (DEGs) related to SMC between SHR and WKY and visualized them using a heat map (Supplementary Figure 1D). Our analysis of scRNA-seq data from AA samples allowed us to identify distinct cell clusters and differentially expressed genes associated with SMC.

### 3.4. Identification of potential core targets for AA rupture leading to CV

A heat map was constructed to demonstrate the 12 common differentially expressed genes associated with AA and CV, including 11 upregulated genes and 1 downregulated gene (Supplementary Figure 2A). To further investigate the expression of these core genes in various cell clusters, we utilized the Seurat package (Supplementary Figure 2B). This study provides insight into potential molecular mechanisms underlying AA and CV and may have implications for future research into disease diagnosis and treatment.

### 3.5. Network construction of differentially expressed miRNAs (DEmiRNAs) and DEGs associated with CV caused by AA

**FIGURE 3:**
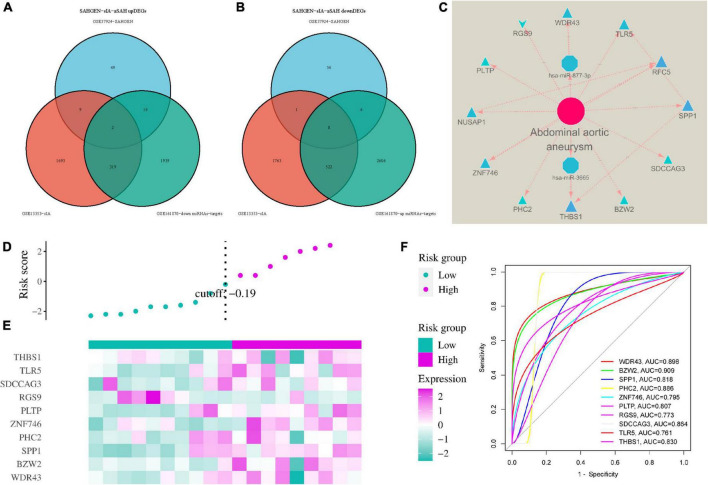
Constructing an interaction network between differentially expressed microRNAs and genes. **(A)** Venn diagram showing co-upregulated target genes. **(B)** Venn diagram showing co-downregulated target genes. **(C)** Protein-protein interaction (PPI) network between DEmiRNAs and mRNAs. **(D,E)** Development of a risk model and evaluation of core genes in cerebral vasospasm (CV) prediction. **(F)** Receiver operating characteristic (ROC) curves of core genes for evaluation of the risk model.

Using differential analysis of GEO data, DEGs were obtained by comparing we obtained DEGs by comparing mRNAs predicted to target binding in DEmiRNAs using differential analysis of GEO data. Based on the negative correlation between miRNA and mRNA expression, we identified two core target potential pathways, hsa-miR-3665/THBS1 and hsa-miR-877-3p/WDR43, that are common target genes (Figures 3A, B). We then created a ceRNA interaction network model of DEmiRNA-mRNA using Cytoscape 3.8.0 software (Figure 3C). By analyzing DEmiRNAs and DEGs associated with CV caused by AA, we identified these two core target potential pathways.

### 3.6. Identification of hub genes for CV

In both CV and AA rupture samples, two genes that were commonly expressed in CV rupture exhibited similar upregulation and downregulation patterns in AA rupture patients. Using these two genes, a disease risk model was constructed to investigate the association between gene expression and disease risk (Figures 3D, E). Patients were stratified into high- and low-risk groups based on their median risk scores. The study found that a greater difference in the expression of the 12 common genes was associated with a higher risk of AA rupture (*P* < 0.05). We generated receiver operating characteristic (ROC) curves to evaluate the sensitivity and specificity of the genes for CV diagnosis (Figure 3F). The area under the curve (AUC) values for nine genes (WDR43, BZW2, SPP1, PHC2, ZNF746, PLTP, RGS9, SDCCAG3, and TLR5) were above 0.70, indicating good diagnostic accuracy of these genes. In summary, our analysis suggests that two genes commonly expressed in CV rupture also exhibit similar expression patterns in AA rupture patients.

### 3.7. Identification of common targets of AA rupture and CV

The 12 common targets of AA rupture and CV were analyzed using the Database for Annotation, Visualization and Integrated Discovery (DAVID). Gene ontology (GO) analysis revealed that the 12 genes were enriched in 273 terms, of which 221 were biological processes (BPs), 14 were cellular components (CCs) and 38 were molecular functions (MFs). These enriched terms included regulation of lipid transport, regulation of lipid localization, response to testosterone, positive regulation of lipid transport, negative regulation of response to wounding and lipid (*P* < 0.05). The results were graphically presented using the “ggplot2” extension package in R Studio software (Supplementary Figures 3A–C). The core targets were found to be related to ECM-receptor interaction, Toll-like receptor signaling pathway, focal adhesion, mismatch repair, phototransduction and DNA replication (Supplementary Figure 3D). The 12 common targets of AA rupture and CV were analyzed using GO analysis and found to be related to ECM-receptor interaction, toll-like receptor signaling pathway, focal adhesion, mismatch repair, phototransduction, and DNA replication.

### 3.8. Screening for drug sensitivity and docking of hub proteins

**FIGURE 4:**
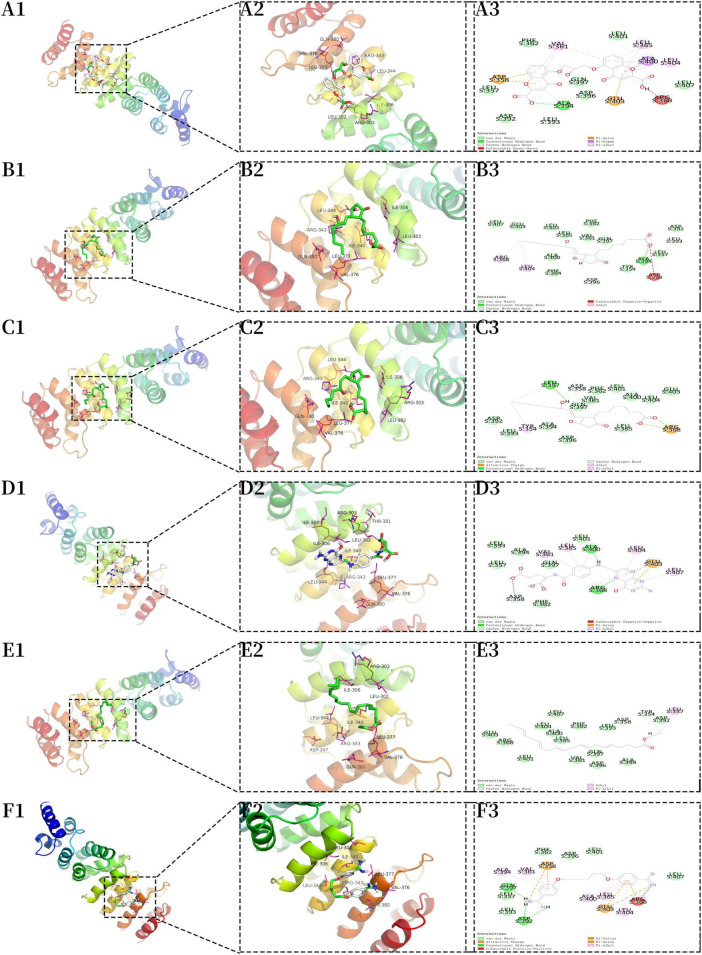
Molecular docking of the core protein WDR43. **(A)** A1, 3D model of WDR43–cromolyn (macroscopic); A2, 3D model of WDR43–cromolyn (microscopic); A3, 2D model of WDR43–cromolyn. **(B)** B1, model of WDR43–edex (macroscopic); B2, 3D model of WDR43–edex (microscopic); B3, 2D model of WDR43–edex. **(C)** C1, 3D model of WDR43–caverject (macroscopic); C2, 3D model of WDR43–caverject (microscopic); C3, 2D model of WDR43–caverject. **(D)** D1, 3D model of WDR43–pga (macroscopic); D2, 3D model of WDR43–pga (microscopic); D3, 2D model of WDR43–pga. **(E)** E1, 3D model of WDR43–epa-e (macroscopic); E2, 3D model of WDR43–epa-e (microscopic); E3, 2D model of WDR43–epa-e. **(F)** F1, 3D model of WDR43–pentamidine (macroscopic); F2, 3D model of WDR43–pentamidine (microscopic); F3, 2D model of WDR43–pentamidine.

We used FDA-approved drugs to dock with the core protein structures of hub proteins WDR43 and THBS1, which were downloaded from the PDB database, in order to identify potential drug candidates. A total of 321 ligands were selected from the NCBI PubChem database. Our analysis identified six compounds–cromolyn, edex, caverject, pga, epa-e, and pentamidine–that specifically bind to the active site of WDR43 with an affinity of <−4.3 kcal/mol. Additionally, we identified six drugs that could bind to the active site of THBS1: lanoxin, dfo, paclitaxel, paromomycin, synribo, and spinosyn D. Using a combination of LibDockScore, binding energy, and RMSD values, we found that cromolyn formed the best docking complex with WDR43, while lanoxin formed the best docking complex with THBS1 (Tables 1, 2, Figure 4, and Supplementary Figure 4).

**TABLE 1:** Docking of WDR43 with the first six compounds.

Protein	Compound	Structure	Vina (kcal/mol)	RMSD	DS (LibDockScore)
WDR43 (7mq8)	Cromolyn	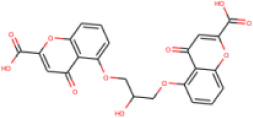	−4.7	2.127	149.071
WDR43 (7mq8)	Edex	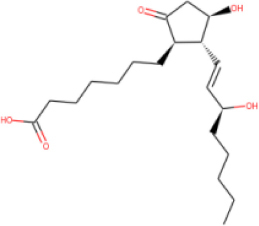	−4.3	2.159	119.099
WDR43 (7mq8)	Caverject	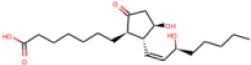	−4.4	1.99	118.899
WDR43 (7mq8)	pga	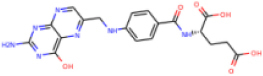	−4.7	3.165	114.992
WDR43 (7mq8)	epa-e	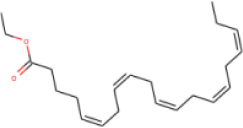	−4.3	1.579	113.211
WDR43 (7mq8)	Pentamidine	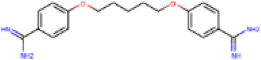	−4.4	1.38	112.552

**TABLE 2:** Docking of THBS1 with the first six Food and Drug Administration (FDA)-approved drugs.

Protein	Compound	Structure	Vina (kcal/mol)	RMSD	DS (LibDockScore)
THBS1 (5FOE)	Lanoxin	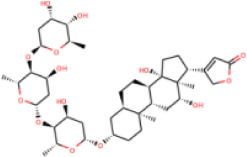	−10.1	1.584	194.557
THBS1 (5FOE)	dfo	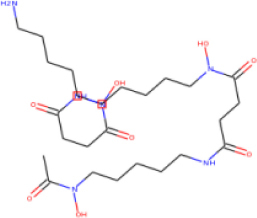	−6.1	1.801	167.336
THBS1 (5FOE)	Paclitaxel	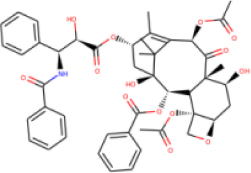	−8.1	3.904	167.13
THBS1 (5FOE)	Paromomycin	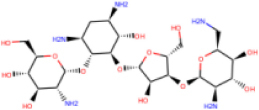	−8.3	2.15	162.305
THBS1 (5FOE)	Synribo	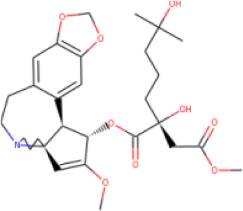	−6.9	2.573	159.613
THBS1 (5FOE)	Spinosyn D	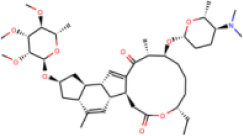	−9.6	2.305	156.037

Of note, cromolyn has the potential to block the hsa-miR-877-3p/WDR43 pathway and may be a promising candidate for preventing CV events after subarachnoid hemorrhage. However, the administration of lanoxin should be approached with caution after cerebral hemorrhage. Our results suggest that these drugs have the potential to target WDR43 and THBS1 and may have implications for future research into disease treatment.

### 3.9. Cromolyn targets WDR43 in molecular dynamics simulations

**FIGURE 5:**
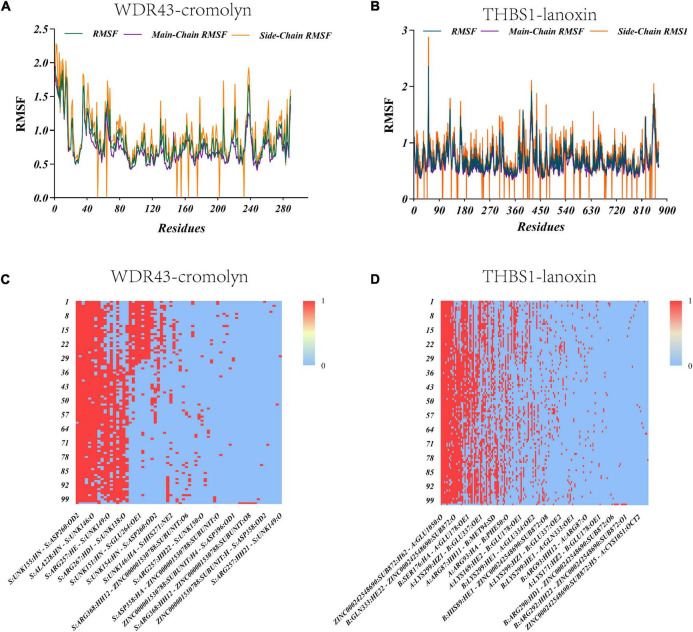
Thermograms demonstrating the root-mean-square fluctuation (RMSF) values and hydrogen bond counts of the two systems. **(A)** Changes in RMSF values of the WDR43-cromolyn complex. **(B)** Changes in RMSF values of the THBS1–lanoxin complex. **(C)** Thermogram demonstrating the hydrogen bonds in the WDR43-cromolyn complex. **(D)** Thermogram demonstrating the hydrogen bonds in the THBS1-lanoxin complex.

Molecular dynamics simulations were performed to investigate the stability of the WDR43-cromolyn and THBS1-lanoxin complexes. The RMSD values for both complexes were calculated over a 100 ns simulation period and found to be within a reasonable range, indicating that the structures of the complexes were in equilibrium (Supplementary Figures 5, 6). The WDR43-cromolyn complex exhibited lower RMSD values compared to the THBS1-lanoxin complex, suggesting greater stability. The RMSF values of all amino acids in the complexes were also calculated (Figures 5A, B). The WDR43-cromolyn complex displayed lower fluctuations around specific amino acid residues, while the THBS1-lanoxin complex showed higher fluctuations around certain amino acid residues. These findings suggest that the WDR43-cromolyn complex is more stable than the THBS1-lanoxin complex. Additionally, a molecular docking thermogram (Figures 5C, D) demonstrated that hydrogen bonds were present in all conformations, particularly in the red columns, indicating their durability. The simulation results of WDR43-cromolyn and THBS1-lanoxin complexes at 25 ns intervals are shown in Supplementary Figure 6. Molecular dynamics simulations were performed to investigate the stability of the WDR43-cromolyn and THBS1-lanoxin complexes, and the WDR43-cromolyn complex was found to be more stable than the THBS1-lanoxin complex.

### 3.10. Cromolyn’s anti-inflammatory function is mediated by WDR43 in BV2 cells

**FIGURE 6:**
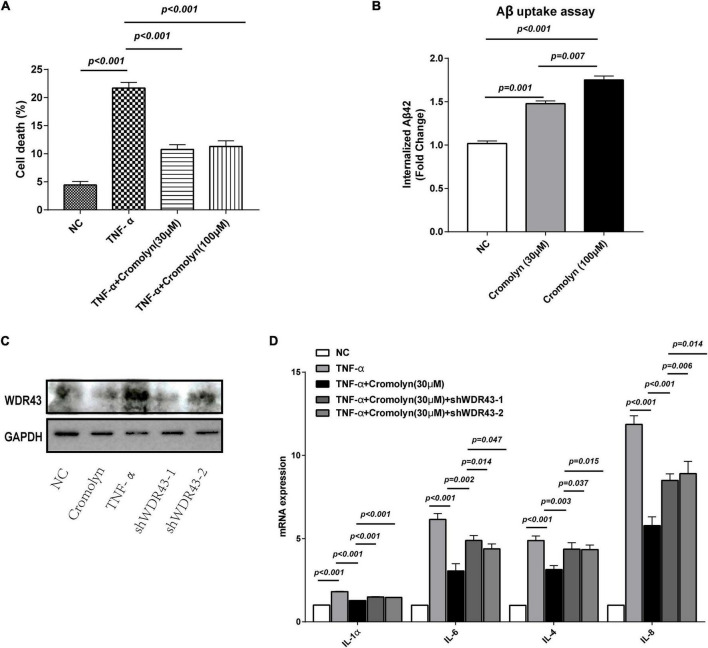
Cromolyn targets WDR43 to exert anti-inflammatory effects on BV2 cells. **(A)** Cell viability assay showed that tumor necrosis factor alpha (TNF)-α induced cell death in BV2 cells in a concentration-dependent manner (0, 0.01, 0.03, 0.1, and 0.3 μg/mL; 24 h). **(B)** Cromolyn promotes the uptake of Aβ42 in BV2 cells: BV2 cells were treated with cromolyn (30 and 100 μM) for 16 h and subsequently incubated with soluble Aβ42 and compounds for 3 h. The cells were collected for ELISA. Aβ42 uptake was higher in BV2 cells treated with cromolyn than in control BV2 cells. **(C)** Western blotting of WDR43 protein extracted from cells treated with cromolyn, TNF-α, shWDR43-1, and shWDR43-2. **(D)** Bar graph demonstrating the mRNA expression of inflammation-related factors in cells treated with cromolyn, TNF-α, shWDR43-1 and shWDR43-2. Data are expressed as the mean ± SEM (*n* = 3; one-way ANOVA).

In BV2 cells, we utilized an *in vitro* model of inflammation to investigate the potential anti-inflammatory function of cromolyn mediated by WDR43 (Wang et al., 2021). Cell viability was assessed by the CCK-8 assay, which revealed that cromolyn impacted the viability of BV2 cells (Figure 6A). Treatment with TNF-α significantly reduced the viability of BV2 cells; However, cromolyn reversed this inhibitory effect. Furthermore, BV2 cells were used to investigate the impact of cromolyn on Aβ42 uptake. Aβ42 uptake was significantly higher in BV2 cells treated with cromolyn (100 μM) compared to vector-treated cells (*n* = 3; Figure 6B).

Transfection with shWDR43-1 and shWDR43-2 resulted in downregulation of WDR43 expression in BV2 cells, while TNF-α treatment increased WDR43 expression (Figure 6C). We measured mRNA expression of inflammation-related factors in cells treated with cromolyn, TNF-α, shWDR43-1, and shWDR43-2 (Figure 6D). Exposure to TNF-α led to an increase in the expression of inflammatory markers in BV2 cells, whereas treatment with cromolyn was able to reverse this effect. Furthermore, downregulation of WDR43 mitigated the effects of cromolyn. These results indicate that cromolyn may rescue the inflammatory phenotype of BV2 cells after TNF-α exposure and enhance Aβ clearance by targeting WDR43. Overall, our findings suggest that cromolyn has potential as an anti-inflammatory drug and may have implications for future research into disease treatment.

## 4. Discussion

Identification of efficacious preventive and therapeutic drugs for secondary dementia following subarachnoid hemorrhage (SAH) and cerebral vasospasm (CV) is of utmost significance. This incapacitating condition can profoundly affect the quality of life for patients and their families and has significant implications for the healthcare system’s economy. Therefore, comprehending the molecular mechanisms underlying CV-induced inflammatory responses in neuronal cells, along with identifying potential therapeutic targets and biomarkers, can expedite drug development and enhance patient outcomes (Amor et al., 2010; Glass et al., 2010; Huang et al., 2022).

In this study, we employed single-cell sequencing, transcriptomic analysis, and molecular docking to identify FDA-approved drugs for preventing secondary dementia after SAH and therapeutic targets for CV. Our findings propose that Cromolyn may forestall dementia by targeting and regulating WDR43 in elderly patients with CV and neuronal inflammatory damage caused by SAH. *WDR43* and *THBS1* are potential therapeutic targets and biomarkers of CV.

Transcriptomic analysis revealed 12 differentially expressed genes (DEGs) shared between AA rupture and CV, of which 11 genes were upregulated (*WDR43, BZW2, SPP1, PHC2, ZNF746, NUSAP1, PLTP, SDCCAG3, TLR5, RFC5, and THBS1*), and one gene was downregulated (RGS9). Gene set enrichment analysis (GSEA) disclosed that these DEGs were enriched in pathways associated with E2F targets, interferon-gamma response, MYC target V2, allograft rejection, complement cascade, and inflammatory response. The E2F transcription factor family plays a pivotal role in determining cell proliferation both intracellularly and extracellularly (Hsu and Sage, 2016). A study reported that *E2F4* is involved in regulating genes associated with AA (Chen et al., 2015). MYC is an oncogene implicated in numerous cellular processes, including cell growth, proliferation, and apoptosis. The MYC target V2 is associated with aggressiveness and poor prognosis of tumors (Schulze et al., 2020). MYC regulates tumor proliferation, metastasis, and metabolism through signaling pathways such as the AKT/mTOR pathway (Li et al., 2016; Xin et al., 2017; Yue et al., 2020). The AKT/mTOR pathway plays a crucial role in the pathophysiology of AA (Wang et al., 2019). Additionally, immune responses and inflammation play a significant role in AA (Shimizu et al., 2006; Sawada et al., 2015). AA is influenced by cytokine release and complement cascade (Hinterseher et al., 2011; Zhang Z. et al., 2018). Reduced C3 concentration and activity in the late-stage of AA may have detrimental effects (Martinez-Pinna et al., 2013). Aortic allografts deficient in interferon signaling can lead to AA associated with an abnormal Th2 pathway (Shimizu et al., 2006). Therefore, the pathways identified through GSEA can help to predict the molecular mechanisms underlying CV-induced inflammatory responses in neuronal cells.

Based on single-cell sequencing data, we identified 28 cell clusters associated with AA, including monocytes, epithelial cells, macrophages, T cells, NK cells, fibroblasts, neutrophils, B cells, and endothelial cells. These findings suggest that AA-associated genes may play an important role in the function of endothelial cells. Specifically, *SPP1* is involved in the transcription of genes in monocytes and production of cytokines in innate immune cells in SLE (Ghodke-Puranik et al., 2021), while PHC2 inhibits interleukin-2 secretion and acts as a negative regulator of Th-cell activity (Cho et al., 2013). Furthermore, expression of BZW2 positively correlates with T cell-mediated immunity to tumor cells and Th2 cells (Galvez et al., 2011; Ge et al., 2022), and THBS1 can reduce the permeability of human dermal microvascular endothelial cells (HDMECs) (Qu et al., 2020). Therefore, we propose that the AA-associated DEGs we identified, such as *SPP1*, *PHC2*, *BZW2*, and *THBS1*, could contribute to inflammatory damage to neuronal cells in CV following SAH.

In addition, using non-transcriptomic data, we identified 406 differentially expressed miRNAs in aortic wall tissue samples from patients with AA and predicted their target mRNAs to core genes associated with AA. We found that two hub genes, *THBS1* and *WDR43*, may serve as potential therapeutic targets for CV following AA. Our risk model and ROC curves indicated that higher differences in the expression of *WDR43* and *THBS1* were associated with higher risk coefficients for the disease, with both genes having an AUC value of 0.70, indicating good diagnostic significance. Therefore, we propose that *WDR43* and *THBS1* may play a critical role in the development of CV.

Recent studies have suggested that the WD repeat protein family member, *WDR43*, may play a role in the development of Parkinson’s disease (PD) and serve as a potential diagnostic biomarker and therapeutic target for PD with comorbid osteoarthritis (Heng et al., 2022). While the exact function of WDR43 remains unclear, it is known to participate in various cellular processes, including transcriptional regulation, cell cycle control, and signal transduction. Dysregulation of protein synthesis and degradation pathways, including the ubiquitin-proteasome system and autophagy-lysosome pathway, has been implicated in the pathogenesis of PD, and previous research has suggested that WDR43 may play a role in ribosome biogenesis and protein synthesis. In addition, WDR43 has been implicated in the development of human estrogen receptor-negative breast cancer and identified as a possible therapeutic target for colorectal cancer (Couch et al., 2016; Bi et al., 2019; Li et al., 2021; Sun et al., 2022). Furthermore, *WDR43* and *NOL11* are required to form a protein complex to ensure successful segregation of chromosomes at kinetochores and centromeres (Shibamura et al., 2004; Fujimura et al., 2020). Although we speculate that WDR43 may be indirectly involved in the development of CV and neurodegeneration following SAH, further research is needed to elucidate its precise mechanisms and potential as a diagnostic biomarker and therapeutic target for these neurodegenerative diseases.

THBS1, on the other hand, plays a significant role in inflammation, angiogenesis, platelet aggregation, tumorigenesis, and tissue remodeling (Silverstein and Febbraio, 2007; Huang et al., 2017; Zhao et al., 2018; Hu et al., 2021; Zhang et al., 2021), and has been extensively studied in lymphoma, breast cancer, melanoma, and gastric cancer (John et al., 2009; Radziwon-Balicka et al., 2014; Zhao et al., 2018; Hu et al., 2021). As a natural inhibitor of angiogenesis, THBS1 can bind to matrix proteins and regulate the activity of MMPs and TIMPs to control extracellular matrix remodeling through membrane receptors in various diseases (John et al., 2009; Radziwon-Balicka et al., 2014; Duan et al., 2021). Platelet-responsive protein-1, whose expression contributes to vascular inflammation by regulating the migration and adhesion of monocytes (Liu et al., 2015), can promote angiotensin II-induced AA in mice when deficient (Krishna et al., 2017). Additionally, THBS1 has been shown to be a key gene associated with abnormal functioning of monocytes/macrophages (Cheng et al., 2022). We postulate that abnormal functioning of monocytes/macrophages after SAH may be associated with the development of CV and neurodegeneration, thus highlighting the potential of THBS1 as a therapeutic target.

Molecular dynamics simulations are widely recognized as reliable tools for investigating protein-ligand interactions (Fu et al., 2020; Kang et al., 2022; Zhang Q. et al., 2022; Zhang Y. et al., 2022). Docking allows for the prediction of the binding pattern and affinity of non-covalent interactions between molecules and is a crucial component of virtual screening (Hassan et al., 2017). Docking allows for the prediction of the binding pattern and affinity of non-covalent interactions between molecules and is a crucial component of virtual screening (Mulpuru and Mishra, 2021). In this study, we utilized molecular docking and MD simulations to investigate the binding of cromolyn to the core protein WDR43, an essential component of the PAF1 complex. Lanoxin was not included in the experiments due to its lack of relevance to the research question. We aimed to evaluate the binding affinity and stability of the cromolyn-WDR43 complex using molecular docking and MD simulations. Our results showed that cromolyn formed the best docking complex with WDR43, indicating a potential therapeutic target for the treatment of cancer. The MD simulations further confirmed the stability of the cromolyn-WDR43 complex, suggesting that it could be a promising lead compound for the development of CV and neurodegeneration.

Cromolyn is known as a “mast cell stabilizer” and exhibits anti-inflammatory effects on various cells, including microglia, which are key regulators of inflammatory responses in the central nervous system (Cox, 1967; Kay et al., 1987; Holian et al., 1991). Based on previous studies, there appears to be some research on the potential role of cromolyn in neurodegeneration, specifically in relation to neuroinflammation. One study found that cromolyn inhibited the secretion of inflammatory cytokines by human microglia, which are key regulators of inflammatory responses in the central nervous system (Zhang C. et al., 2018; Kwon and Koh, 2020). Another study suggests that cromolyn, although its mechanism of action is not well-understood, has been known for some time as a mast cell stabilizer, and may have potential therapeutic value in neurodegenerative diseases (Wang et al., 2021). Therefore, cromolyn is known as a “mast cell stabilizer” and exhibits anti-inflammatory effects on various cells (Granucci et al., 2019) and has potential neuroprotective effects in neurodegenerative diseases by acting on microglia (Hori et al., 2015; Kondo et al., 2017). However, it is important to note that the research on the relationship between cromolyn and neurodegeneration is still in its early stages, and more research is needed to fully understand its potential benefits and limitations in this context. Additionally, it is important to discuss any potential use of cromolyn for the treatment of neurodegenerative diseases with a healthcare professional, as cromolyn is currently only approved for the treatment of asthma. Our study provides evidence that cromolyn could exert neuroprotective effects by targeting WDR43, suggesting that WDR43 and cromolyn are promising targets for the treatment of CV and neurodegeneration.

This study aimed to investigate the genetic mechanisms underlying dementia caused by CV in the elderly and identify potential preventive and therapeutic drugs. While our study identified 11 upregulated genes, including *WDR43* and *THBS1*, and one downregulated gene associated with aneurysm rupture, we acknowledge the limitations of not conducting relevant experiments on miRNA. In recent years, miRNAs have gained increasing attention due to their crucial regulatory roles in disease pathogenesis and progression. The identification of miRNAs associated with different diseases and the elucidation of their regulatory mechanisms provide important insights into disease pathogenesis and potential therapeutic interventions. Overall, miRNAs have become essential tools in the study and treatment of various diseases, highlighting their importance and promising roles in safeguarding human health. We plan to conduct relevant experiments on miRNA in our future research to further enhance our understanding of the molecular mechanisms involved in the development of CV-induced dementia.

While our study identified key biomarkers associated with dementia following SAH and CV, we acknowledge the limitations of not conducting relevant experiments on miRNA. Further research is needed to fully explore the regulatory roles of miRNAs in disease pathogenesis and progression and their potential as therapeutic targets for neurodegenerative disorders. Additionally, experimental validation is required to confirm the findings of this study and explore the mechanisms of action of identified drugs. Nonetheless, our study provides important insights into the molecular mechanisms involved in the development of CV-induced dementia and has the potential to advance drug development for the treatment of CV and neurodegeneration following SAH.

## 5. Conclusion

This study has identified WDR43 and THBS1 as potential therapeutic targets and genetically relevant markers for CV occurring after SAH. Moreover, our findings have demonstrated that cromolyn exerts neuroprotective effects by targeting WDR43, which is a novel finding that suggests the potential use of cromolyn for the treatment of CV and neurodegeneration. These results provide important insights into the genetic mechanisms underlying the development of CV and neurodegeneration after SAH and offer a theoretical groundwork for further research in this field.

## Data availability statement

Publicly available datasets were analyzed in this study. This data can be found in the GEO repository here: accession numbers GSE161870 (https://www.ncbi.nlm.nih.gov/geo/query/acc.cgi?acc=GSE161870), GSE37924 (https://www.ncbi.nlm.nih.gov/geo/query/acc.cgi?acc=GSE37924), and GSE13353 (https://www.ncbi.nlm.nih.gov/geo/query/acc.cgi?acc=GSE13353).

## Author contributions

XW, ZL, and FK performed the data curation and analysis, analyzed and interpreted the results, and drafted and reviewed the manuscript. All authors read and approved the final manuscript.

## Footnotes

1
https://cn.string-db.org/

2
http://www.interactivenn.net/

3
http://www.rcsb.org/

## Supplementary material

SUPPLEMENTARY FIGURE 1 Identification of differentially expressed microRNAs associated with ruptured aortic aneurysms. **(A)** Heat map showing the differentially expressed microRNAs (DEmiRNAs) associated with ruptured aortic aneurysms (AA) in the GSE161870 dataset. **(B)** Volcano plot depicting the distribution of DEmiRNAs in the GSE161870 dataset. **(C)** Predicted target genes of upregulated DEmiRNAs. **(D)** Predicted target genes of downregulated DEmiRNAs.

SUPPLEMENTARY FIGURE 2 Identification of overlapping genes between cerebral vasospasm (CV) and aortic aneurysm rupture. **(A)** Heat map of AA-associated cell clusters. **(B)** Expression levels of 12 genes evaluated based on single-cell data.

SUPPLEMENTARY FIGURE 3 Results of gene ontology (GO) and Kyoto Encyclopedia of Genes and Genomes (KEGG) pathway enrichment analyses. **(A)** GO/biological process analysis of hub genes. **(B)** GO/cellular component analysis of hub genes. **(C)** Chord diagram demonstrating the results of GO/molecular function analysis of hub genes. **(D)** Chord diagram demonstrating the results of GO/KEGG pathway analysis.

SUPPLEMENTARY FIGURE 4 Molecular docking of the core protein THBS1. (**A**) A1, 3D model of THBS1–lanoxin (macroscopic); A2, 3D model of THBS1–lanoxin-3D (microscopic); A3, 2D model of THBS1–lanoxin. **(B)** B1, 3D model of THBS1–dfo (macroscopic); B2, 3D model of THBS1–dfo (microscopic); B3, 2D model of THBS1–dfo. **(C)** C1, 3D model of THBS1–paclitaxel (macroscopic); C2, 3D model of THBS1–paclitaxel (microscopic); C3, 2D model of THBS1–paclitaxel. **(D)** D1, 3D model of THBS1–paromomycin (macroscopic); D2, 3D model of THBS1–paromomycin (microscopic); D3, 2D model of THBS1–paromomycin. **(E)** E1, 3D model of THBS1–synribo (macroscopic); E2, 3D model of THBS1–synribo (microscopic); E3, 2D model of THBS1–synribo. **(F)** F1, 3D model of THBS1–spinosyn D (macroscopic); F2, 3D model of THBS1–spinosyn D (microscopic); F3, 2D model of THBS1–spinosyn D.

SUPPLEMENTARY FIGURE 5 RMSD value of the system during molecular dynamics (MD) simulation. Molecular dynamics simulations for the WDR43–cromolyn and THBS1–lanoxin complexes are shown in blue and orange, respectively.

SUPPLEMENTARY FIGURE 6 Molecular dynamics simulations of conformational changes in docked complexes. **(A)** Simulation results of the WDR43-cromolyn complex at 25 ns intervals. **(B)** Simulation results of the THBS1-lanoxin complex at 25 ns intervals.
