# Supplementary figures and images for: Cromolyn prevents cerebral vasospasm and dementia by targeting WDR43
Source: Front Aging Neurosci. 2023 Apr 13;15:1132733. doi: 10.3389/fnagi.2023.1132733 (PMC10133528; doi:10.3389/fnagi.2023.1132733)

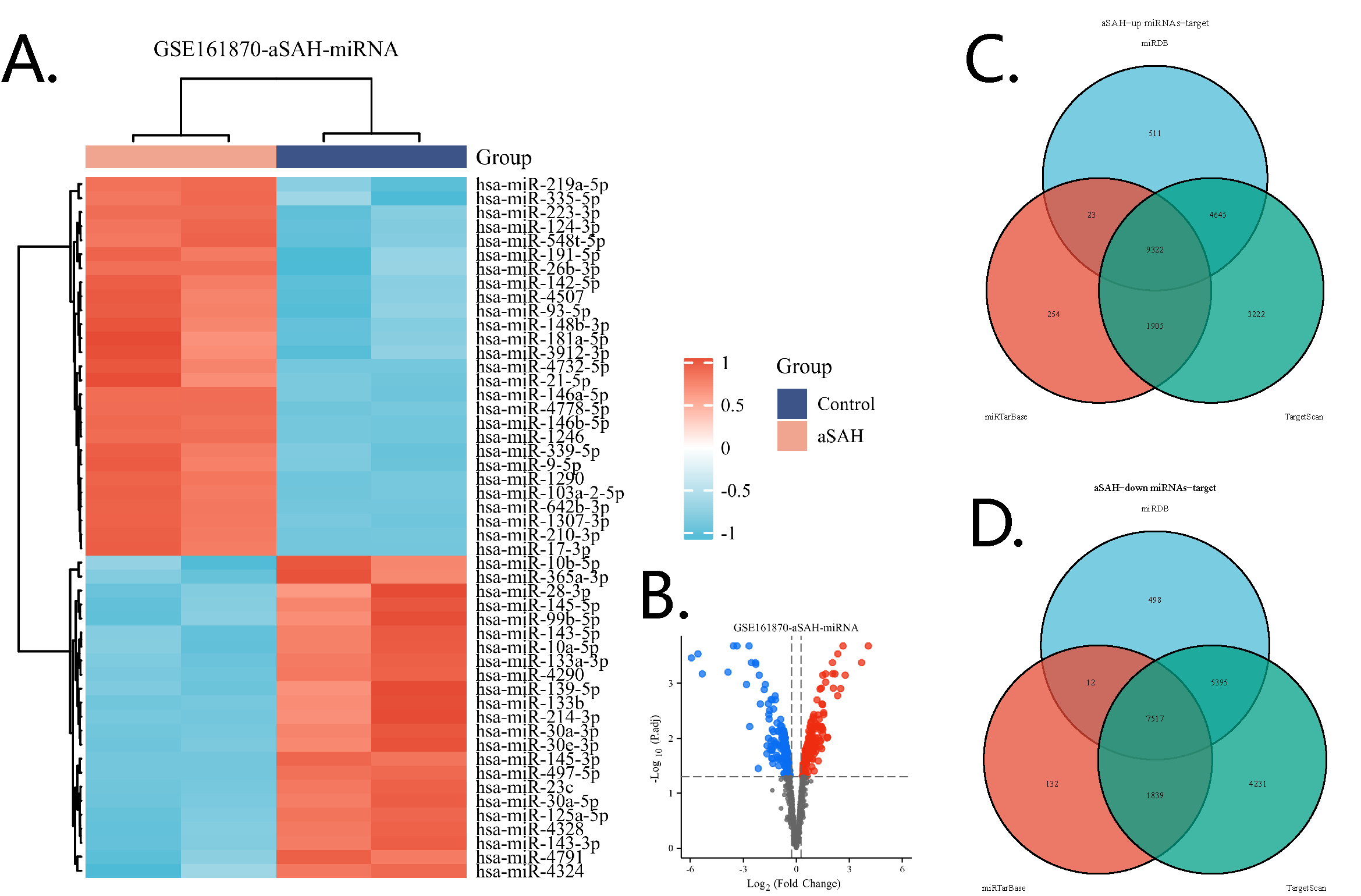

Supplement: SUPPLEMENTARY FIGURE 1 — Identification of differentially expressed microRNAs associated with ruptured aortic aneurysms. (A) Heat map showing the differentially expressed microRNAs (DEmiRNAs) associated with ruptured aortic aneurysms (AA) in the GSE161870 dataset. (B) Volcano plot depicting the distribution of DEmiRNAs in the GSE161870 dataset. (C) Predicted target genes of upregulated DEmiRNAs. (D) Predicted target genes of downregulated DEmiRNAs. [file Image_1.TIF]

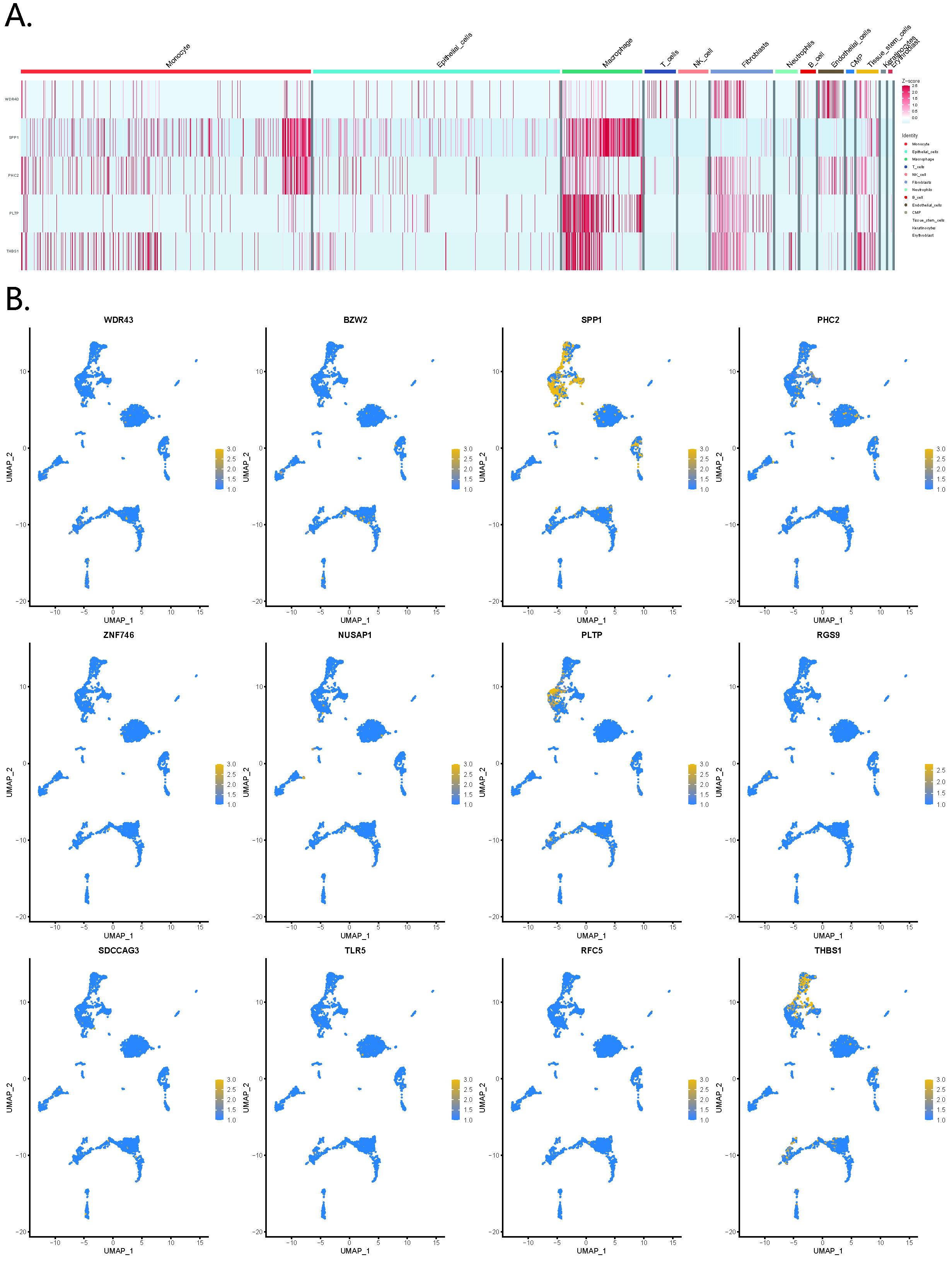

Supplement: SUPPLEMENTARY FIGURE 2 — Identification of overlapping genes between cerebral vasospasm (CV) and aortic aneurysm rupture. (A) Heat map of AA-associated cell clusters. (B) Expression levels of 12 genes evaluated based on single-cell data. [file Image_2.TIF]

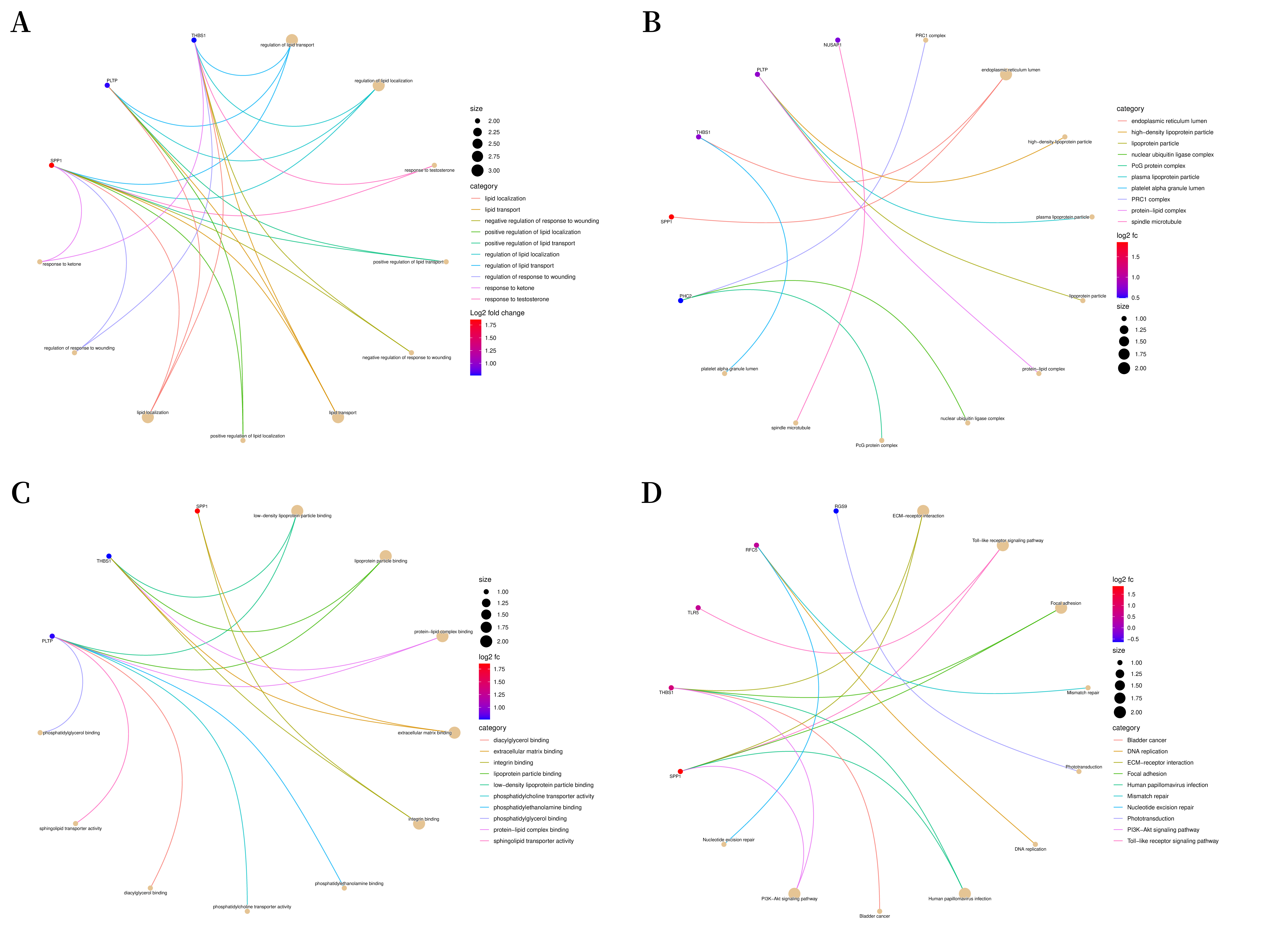

Supplement: SUPPLEMENTARY FIGURE 3 — Results of gene ontology (GO) and Kyoto Encyclopedia of Genes and Genomes (KEGG) pathway enrichment analyses. (A) GO/biological process analysis of hub genes. (B) GO/cellular component analysis of hub genes. (C) Chord diagram demonstrating the results of GO/molecular function analysis of hub genes. (D) Chord diagram demonstrating the results of GO/KEGG pathway analysis. [file Image_3.TIF]

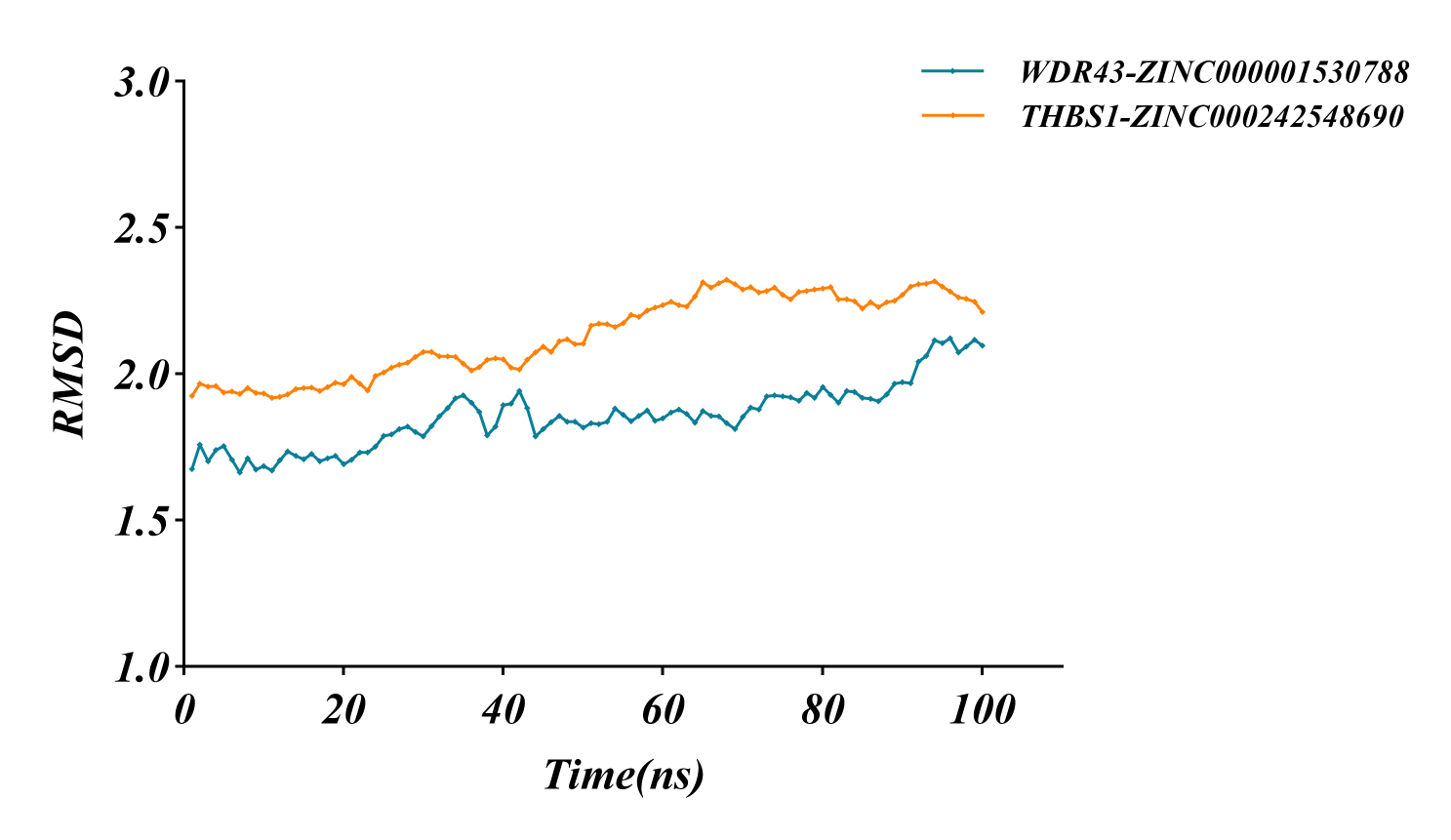

Supplement: SUPPLEMENTARY FIGURE 5 — RMSD value of the system during molecular dynamics (MD) simulation. Molecular dynamics simulations for the WDR43–cromolyn and THBS1–lanoxin complexes are shown in blue and orange, respectively. [file Image_5.TIF]
